# Evolving strategy and long-term surgical outcome of mitral valve repair in congenital mitral valve stenosis

**DOI:** 10.1186/1749-8090-8-S1-O273

**Published:** 2013-09-11

**Authors:** Eva Delmo Walter, Roland Hetzer

**Affiliations:** 1 Cardiothoracic Surgery, Deutsches Herzzentrum Berlin, Berlin, Germany

## Background

Having collected a large series of congenital mitral stenosis we studied the operative results and long-term outcome of our evolving mitral valve (MV) repair techniques performed to correct this lesion.

## Methods

Between 1986 and 2012, 137 infants and children (mean age 4.1±5.0 (range 1 month to 16.8 years) underwent surgical correction of congenital mitral stenosis (CMS). In 48 patients, CMS is involved in Shone’s anomaly. The typical congenital MS (Type I) was seen in 56 patients., hypoplastic MV (Type II) in 15 patients,supravalvar mitral ring (Type III in 48 patients, parachute MV (Type IV) in 10 patients and hammock valve (Type IV) in 8 patients.

## Results

MV repair was performed using commissurotomy, division of chordae tendinae, papillary muscle splitting and fenestration, and resection of mitral ring, applied according to the presenting morphology in patients with either previously corrected or concomitant correction of the left-sided obstructive lesions. Postoperative echocardiography showed absence of MV stenosis and immediate improvement of symptoms, except in a 3-month-old infant who died 18 days postoperatively due to myocardial failure. During the 24-year follow-up, 23 patients underwent repeat MV repair and 3 underwent MV replacement after failed attempts at repair. Mean duration of follow-up was 17.5±1.5 years. Freedom from reoperation was 97.6±2.4%, 89.3±5.1%, and 52.8±11.8%, at 30 days, 1 and 15 years postoperatively, respectively Cumulative survival rate was 97.6±2.4%, 92.3±4.3%, and 70.3±8.9%, at 30 days, 1 and 15 years postoperatively, respectively Mortality unrelated to valve repair accounted for 9 (20%) deaths.

## Conclusions

Long-term functional outcome of mitral valve repair in children with CMS is satisfactory. Repeat MV repair and/or replacement may be deemed necessary during the course of follow-up.

